# Mitotic Slippage and Expression of Survivin Are Linked to Differential Sensitivity of Human Cancer Cell-Lines to the Kinesin-5 Inhibitor Monastrol

**DOI:** 10.1371/journal.pone.0129255

**Published:** 2015-06-02

**Authors:** Hila Asraf, Rachel Avunie-Masala, Michal Hershfinkel, Larisa Gheber

**Affiliations:** 1 Department of Physiology and Cell Biology, Faculty of Health Sciences, Ben-Gurion University of the Negev, Beer-Sheva, Israel; 2 Department of Chemistry, Faculty of Natural Sciences, Ben-Gurion University of the Negev, Beer-Sheva, Israel; 3 Ilse Katz Institute for Nanoscale Science and Technology, Ben-Gurion University of the Negev, Beer-Sheva, Israel; Fudan University, CHINA

## Abstract

The mitotic Kinesin-5 motor proteins crosslink and slide apart antiparallel spindle microtubules, thus performing essential functions in mitotic spindle dynamics. Specific inhibition of their function by monastrol-like small molecules has been examined in clinical trials as anticancer treatment, with only partial success. Thus, strategies that improve the efficiency of monastrol-like anticancer drugs are required. In the current study, we examined the link between sensitivity to monastrol and occurrence of mitotic slippage in several human cell-lines. We found that the rank of sensitivity to monastrol, from most sensitive to least sensitive, is: AGS>HepG2>Lovo>Du145≥HT29. We show correlation between the sensitivity of a particular cell-line to monastrol and the tendency of the same cell-line to undergo mitotic slippage. We also found that in the monastrol resistant HT29 cells, prolonged monastrol treatments increase mRNA and protein levels of the chromosomal passenger protein survivin. In contrast, survivin levels are not increased by this treatment in the monastrol-sensitive AGS cells. We further show that over-expression of survivin in the monastrol-sensitive AGS cells reduces mitotic slippage and increases resistance to monastrol. Finally, we show that during short exposure to monastrol, Si RNA silencing of survivin expression reduces cell viability in both AGS and HT29 cells. Our data suggest that the efficiency of anti-cancer treatment with specific kinesin-5 inhibitors may be improved by modulation of expression levels of survivin.

## Introduction

The mitotic Kinesin-5 motor proteins (BimC/Kif11/Eg5/N-2) perform conserved functions in mitotic spindle dynamics. Discovered in the early 1990s, these were the first kinesins for which mitotic roles have been demonstrated in a number of organisms [1–5]. Kinesin-5 motors function as homotetramers with two pairs of catalytic motor domains located at opposite sides of a dumbbell-like tetrameric complex [6, 7]. By this bipolar structure, kinesin-5 motors can crosslink and slide apart antiparallel spindle microtubules [8–11], thus performing their functions in spindle assembly [1–5] and anaphase spindle elongation [12–19].

The human kinesin-5 HsEg5 is overexpressed in a variety of solid tumors, suggesting its role in tumorigenesis [20, 21]. Because of the essential mitotic functions of kinesin-5 motors in spindle dynamics, and because mitosis is an accepted cell-cycle phase for anti-cancer intervention [22, 23], it was generally believed that specific inhibition of kinesin-5 motors could serve as a potential anti-cancer treatment. Monastrol was the first reported specific inhibitor of human kinesin-5, identified in a screen for small molecules that caused mitotic arrest without affecting microtubule dynamics and other cellular functions [24]. Since the discovery of monastrol, several tens of molecules were reported as allosteric inhibitors of HsEg5, with variable potencies [23, 25]. The majority of these molecules are specific for the human HsEg5 because they bind to an allosteric site, loop 5 in the catalytic domain of kinesin-related motors (reviewed in [23, 26, 27]), which varies in length and sequence among the kinesin homologues [28, 29]. Human cells treated with monastrol and monastrol-like molecules arrest in mitosis with damaged monopolar spindles [24, 30] and undergo mitotic cell death [31]. In some cases monastrol treated cells are found in a G1-like phase due to mitotic slippage [32]. The latter phenomenon allows cells to proceed to the next G1 phase without dividing their DNA in the presence of spindle damage (reviewed in [33, 34]). Following mitotic slippage, cells can die of apoptosis caused by a specific checkpoint that monitors the DNA content of cells that exit mitosis, known as the "tetraploidy checkpoint" [33, 35].

Several specific HsEg5 inhibitors have entered clinical trials as anticancer agents [36–38]. In spite of the reproducible cytotoxic effect in tissue cultures, these clinical trials revealed limited success (reviewed in [27, 39]). One of the proposed reasons for this inefficiency is incomplete knowledge of the mitotic arrest pathways and, as a result, inability to identify molecular components that can be targeted in addition to kinesin-5 inhibitors to improve their efficiency in anticancer treatment [27, 39].

To address this issue, in the current study we examined the sensitivity to monastrol and occurrence of mitotic slippage in several human cell-lines. We found that there is a correlation between the sensitivity of a particular cell-line to monastrol and the tendency of the same cell-line to undergo mitotic slippage. We further examined the expression of survivin, an anti-apoptotic chromosomal passenger protein that has been demonstrated to have multiple mitotic roles (reviewed in [40–43]). We found that treatment with monastrol induces increase in the expression of survivin in monastrol-resistant cells, but not in cells that are monastrol-sensitive. Consistently, we show that over-expression of survivin in the monastrol-sensitive cells reduced mitotic slippage and increased monastrol-resistance. Finally, we show that partial silencing of survivin expression by Si RNA reduces cell viability following short exposure to monastrol. Thus, our data suggest that combined inhibition of HsEg5 and modulation of survivin expression can improve the potency of anticancer treatment by kinesin-5 inhibitors.

## Materials and Methods

### Cell culture, viability, transfection, and monastrol treatment

**Fig 1:**
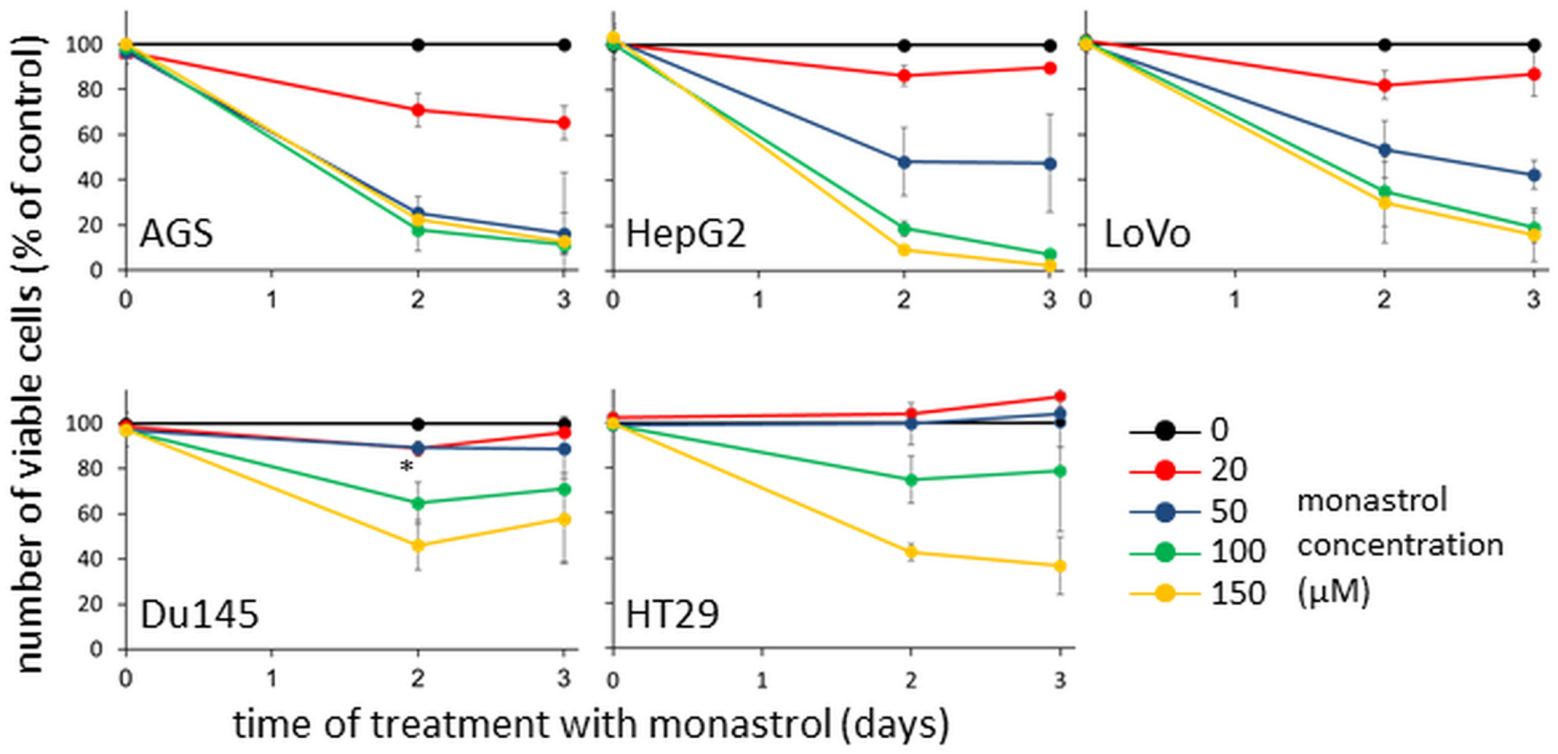
Effect of prolonged treatment with monastrol on cell viability. Cells from AGS, HepG2, Lovo39, Du145, and HT29 cell lines, indicated at the lower left corner of each panel, were incubated for up to three days with various concentrations of monastrol, indicated on the right (0, 20, 50, 100, and 150μM). The number of viable cells (% of control) was determined by XTT assay for mitochondrial activity. Points and bars represent an average and SEM of 3–4 independent experiments. *P<0.05: the number of viable Du145 cells following 2-day treatment with 20 or 50μM monastrol, compared to viable HT29 cells identically treated (red and blue circles). The results indicate that the different cell lines are differently sensitive to monastrol, with the sensitivity ranking: AGS>HepG2>Lovo>Du145≥HT29.

AGS and LoVo cells were grown in DMEM/F-12 (HAM) 1:1, HT29 cells in DMEM, Du145 cells in RPMI 1640 supplemented with 1% Sodium Pyruvate, and HepG2 cells in MEM-EAGLE Earle's medium. All media were supplemented with 10% fetal calf serum, 2mM L-glutamine, and 1% antibiotic-antimycotic solution containing 10 units/μl penicillin, 10 μg/μl streptomycin, and 1250 units/ml nystatin. Media reagents were purchased from Beit Haemek, Israel. Cell viability was examined using sodium 2,3-Bis-(2-methoxy-4-nitro-5-sulfophenyl)-2H-tetrazolium-5-carboxanilide salt (XTT, Sigma, Israel), following a standard procedure [44] or by Trypan blue assay [30]. For viability experiments, up to 3-days using the XTT assay, ~4x10^3^ cells were plated per well in 96-well plates. Number of cells in the presence of monastrol was normalized to the number of cells in the presence of DMSO only. For experiments using 12 and 24h monastrol treatment, ~1.5x10^5^ cells per well were plated in 24-well plates. Treatment with monastrol was carried out 24h following plating, at which time 70–80% confluence was achieved. Plasmids expressing GFP-tagged survivin and GFP vector only were a gift from Dr. Dario C. Altieri [45]. Cell transfection was performed using the JetPEI transfection reagent (Polyplus transfection, Tal Ron, Israel). Stable transfection was achieved by growing cells in medium containing G418 3 mg/ml (Sigma, Israel) for several weeks until 80% of cells contained GFP fluorescence signal. Monastrol (Tocris, UK) concentrations are indicated for each experiment. For comparison between monastrol-sensitive AGS and monastrol-resistant HT29 cells, different concentrations of monastrol were used to achieve comparable phenotypes: 100μM and 150μM of monastrol for AGS and HT29 cells, respectively. Based on Fig 1, low concentration of monastrol would have resulted in lack of effect on the HT29 cells, while high concentration of monastrol would have resulted in death of the AGS cells, either case not allowing analysis of the phenotype. Control experiments were performed in the presence of DMSO.

### Immunostaining

To visualize the microtubule cytoskeleton and DNA, cells were cultured on coverslips, fixed with 4% paraformaldehyde for 30 min, and permeabilized for 2 min with 0.3% Triton-x-100 in 4% paraformaldehyde. Samples were washed with PBS containing 0.1% BSA (Sigma, Israel). Coverslips were incubated with rat anti-tubulin YOL1/34 and then with Alexa 488 conjugated anti-rat secondary antibody. DNA was stained by DAPI (0.07 μg/ml). Cells were observed with an Olympus BX51 microscope.

### Cell cycle analysis

Floating and adherent cells were fixed with 70% ethanol and stored in -20°C for at least 7 days. Cells were collected by centrifugation and resuspended in 0.1% Triton-X-100 and 30 μg/ml RNAse A type I-A (Sigma, Israel) at room temperature for 40 min. Nuclear DNA was stained with 15 μg propidium iodide in PBS solution and DNA content was measured by flow cytometry (BD Biosciences, UK). Cell proportions in sub-G0/G1, G0/G1, S, and G2/M phases were analyzed using appropriate software. For each sample, 10,000 cells were scored.

### mRNA analysis

Levels of Cyclin B, survivin, and actin as a reference gene were determined by real time PCR (RT-PCR) of total RNA extracted from cells treated with monastrol. Total RNA extraction was performed with the RNeasy mini kit (Qiagen, Germany), with DNAse (Qiagen, Germany) treatment to ensure removal of genomic DNA. The cDNA template was prepared with 1 μg RNA samples, using the Verso cDNA Synthesis Kit as described by the manufacturer (Thermo Scientific). For quantitative real time PCR, templates were diluted 1:16 and subjected to Taqman real time PCR procedure using the ABsolute Blue QPCR kit (Thermo Scientific). Primers and probes (Solaris) for PCR amplification and product size were as follows: survivin (95 bp) forward primer 5'-TTTCTCAAGGACCACCGCAT-3', reverse primer 5'-ATGAAGCCAGCCTCGGCCAT-3', probe 5'-CACCCCGGAGCGGATGG-3'; Cyclin B (86 bp) forward primer 5'-ATCTGAGACAACTTGAGGAAG-3', reverse primer 5'-GATGGCTCTCATGTTTCCAG-3', probe 5'-GGTCGGGAAGTCACTGG-3'; Actin (134 bp) forward primer 5'-TGGAGAAAATCTGGCACCAC-3', reverse primer 5'-GGTCTCAAACATGATCTGG-3', probe 5'-ACCGCGAGAAGATGACC-3'. Reactions were carried out in the ABI-PRISM7500 sequence detector (Applied Biosystems, Darmstadt, Germany). Standard cycling conditions for this instrument were used. Annealing temperature was 60°C for all the genes. For each sample, the levels of cyclin B and survivin were normalized to the reference gene (actin) levels. Real-time PCR results represent an average of three different experiments.

Protein level analysis was performed using Western blot (WB) procedure as previously described [30], using anti human survivin antibody (R&D Systems), cyclin B1 (Santa Cruz Biotechnology Inc.), or β-actin (Cell Signaling Technology, Inc. MA, USA). Densitometric analysis of protein expression level was performed using EZQuant-Gel image processing and analysis software (EZQuant, Rehovot, Israel). Protein levels were normalized to actin levels.

### Si RNA silencing

Cells were plated at ~1.5x10^5^ cells per well in 24 well plates; monastrol was applied 24h following plating. Cells were transfected with siSurvivin or a scrambled (SC) siRNA construct (40nM, Sigma-Aldrich), siRNA transfection was performed using the lipofectamine 2000 reagent according to the manufacturer’s protocol. The target sequence of the survivin for siRNA was: 5' GGACCACCGCAUCUCUACA 3' or scrambled 5' GCCCAGAUCCCUGUACGU 3'. 12h and 24h post transfection cells were counted using Trypan blue.

### Statistical analysis

Columns and bars in all figures represent average ± SEM. The significance of the differences between the averaged values was determined using Student’s *t*-test. *P<0.05; **P<0.01.

## Results

To examine the factors that influence the sensitivity of different cells to monastrol, we first characterized this sensitivity in five different cell lines: AGS from stomach adenocarcinoma [46], HepG2 from hepatocellular carcinoma [47], LoVo from colon adenocarcinoma [48], Du154 from prostate carcinoma [49], and HT29 from colon adenocarcinoma [50]. Cells were subjected to prolonged treatment with monastrol of up to three days. At each time interval, the number of viable cells was examined by mitochondrial activity assay using 2,3-Bis-(2-methoxy-4-nitro-5-sulfophenyl)-2H-tetrazolium-5-carboxanilide salt (XTT) [44]. Our results indicate that, as expected, the number of viable cells decreased following prolonged treatment with monastrol in all cell lines. However, the percentage of viable cells in the different cell lines following treatment with monastrol varied. For example, treatment with 100μM of monastrol for two days resulted in ~80% decrease in numbers of AGS and HepG2 cells, ~60% decrease in number of Lovo cells, and only ~30% decrease in Du145 and HT29 cells (Fig 1, green circles). We also found a small but reproducible difference between the viability of Du145 and HT29 cells treated with monastrol. For example: the percent of viable Du145 cells treated with 20 or 50μM monastrol for 2 days is significantly smaller than that of the identically treated HT29 cells. Based on the viability data (Fig 1), we established the rank of sensitivity to monastrol, from most sensitive to least sensitive, as: AGS>HepG2>Lovo>Du145≥HT29.

**Fig 2:**
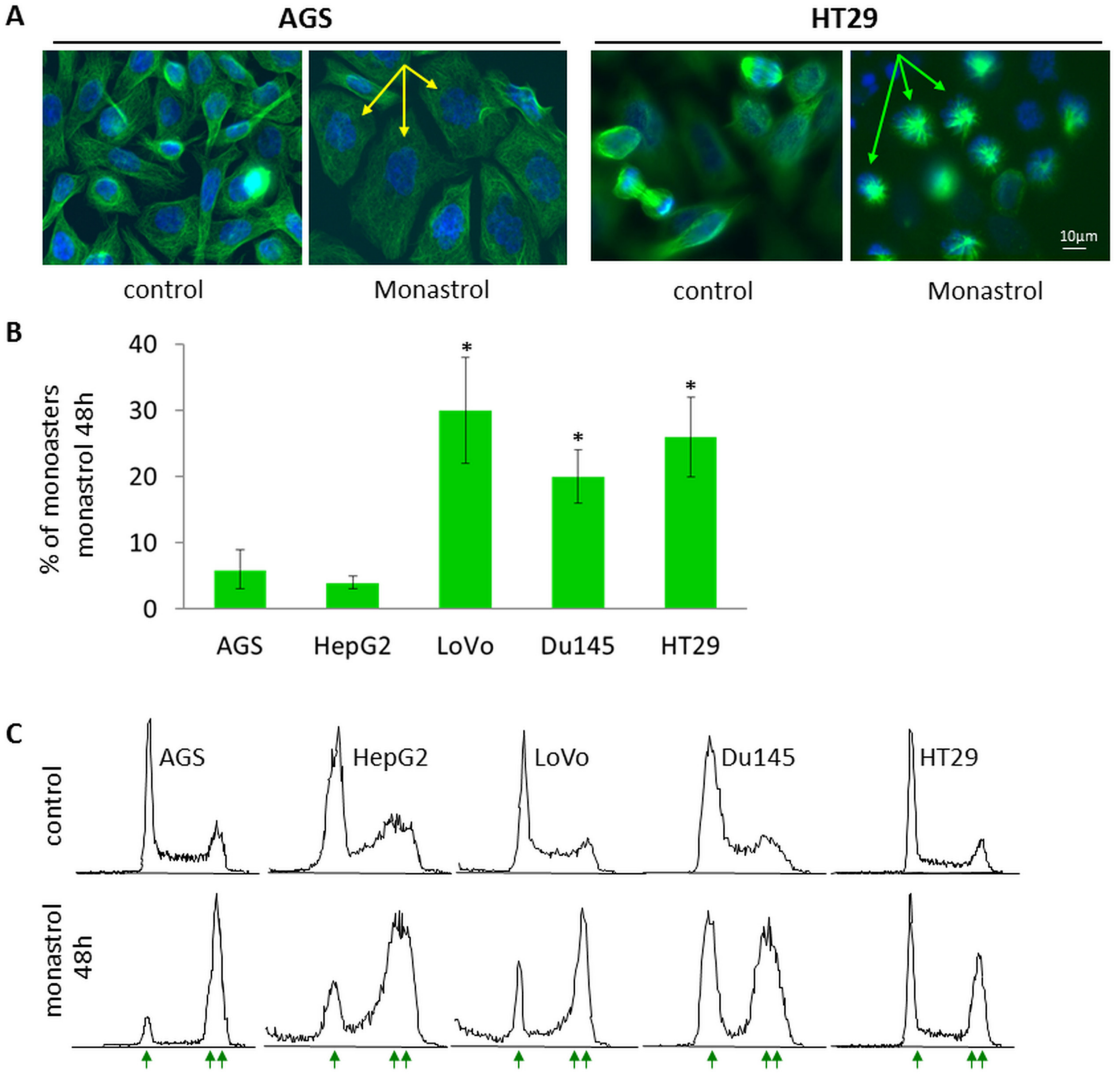
Microtubule cytoskeleton and cell morphology, and cell-cycle distribution following 48h treatment with monastrol. (A) AGS and HT29 cells were treated for 48h with 100μM and 150μM of monastrol, respectively. Tubulin cytoskeleton was visualized following fixation by immunostaining, DNA was stained with DAPI. Following 48h of monastrol treatment, the majority of AGS cells appeared as large, G1-like cells with one nucleus (yellow arrows), while a high percentage of HT29 cells were arrested in mitosis as monoasters (green arrows). (B) Effect of prolonged treatment with monastrol on percentage of monoasters in the different cell-lines, indicated at the bottom. Cells were treated with monastrol, processed for immunostaining, and the percentage of monoasters was determined by scoring 200–300 cells for each cell-line. Columns and bars represent averages and SEM of 3 independent experiments, *P<0.05, compared to AGS cells. (C) Distribution of cell-cycle phases was determined by flow cytometry in the absence (control) or presence of monastrol. Cell-lines are indicated on top. Single and double arrows represent peaks with 2n and 4n DNA content, respectively.

Mitotic slippage is a phenomenon by which in the presence of mitotic damage, cells proceed to the G1 phase of the next cycle without dividing their DNA [31, 33, 34, 51]. We next examined whether the resistance of a particular cell line to monastrol is related to its tendency to undergo mitotic slippage in the presence of the drug. To characterize mitotic slippage, we treated cells with monastrol for 48h at concentrations that resulted in least cell survival, and examined the morphology of microtubule cytoskeleton by immunostaining (Fig 2A and 2B) and cell-cycle distribution by flow cytometry (Fig 2C). Cells arrested in mitosis by monastrol have previously been shown to exhibit monoastral spindles [24, 30]. We found that following 48h monastrol treatment two main morphologies of cells were apparent: G1-like or monoastral mitotic cells (Fig 2A). The number of normal mitotic cells or cells with unidentified morphologies was negligible. Our results show that following 48h treatment with monastrol, the different cell-lines exhibited different percentages of cells with monoasters. For example, the majority of surviving AGS cells appeared as G1-like large cells with single nuclei (Fig 2A). The percentage of monoasters in these cells was very small, ~7% (Fig 2B). We found that both AGS and HepG2 cell-lines, which are sensitive to monastrol, exhibited a small percentage of monoasters (<10%) and a large percentage of G1-like cells, following 48h monastrol treatment (Fig 2B). On the other hand, LoVo, Du145, and HT29 cell-lines, which are more resistant to monastrol, exhibit considerably larger percentages, 20–30%, of monoastral spindles (Fig 2B), indicating that large numbers of these cells are arrested in mitosis. Analysis of the cell-cycle phase distribution revealed that following 48h treatment with monastrol, in spite of the difference in percentage of mitotic cells with monoasters, all examined cell-lines contained a large percentage of cells with double DNA content (Fig 2C, double arrow). This indicates that although some of the cells appeared with the typical G1-like microtubule cytoskeleton, the cells had doubled DNA content, i.e., they underwent mitotic slippage without DNA division. Our results therefore suggest that there is a link between the sensitivity to monastrol and the tendency to undergo mitotic slippage: monastrol-resistant cells hold mitotic arrest for longer times, while monastrol-sensitive cells undergo mitotic slippage.

**Fig 3:**
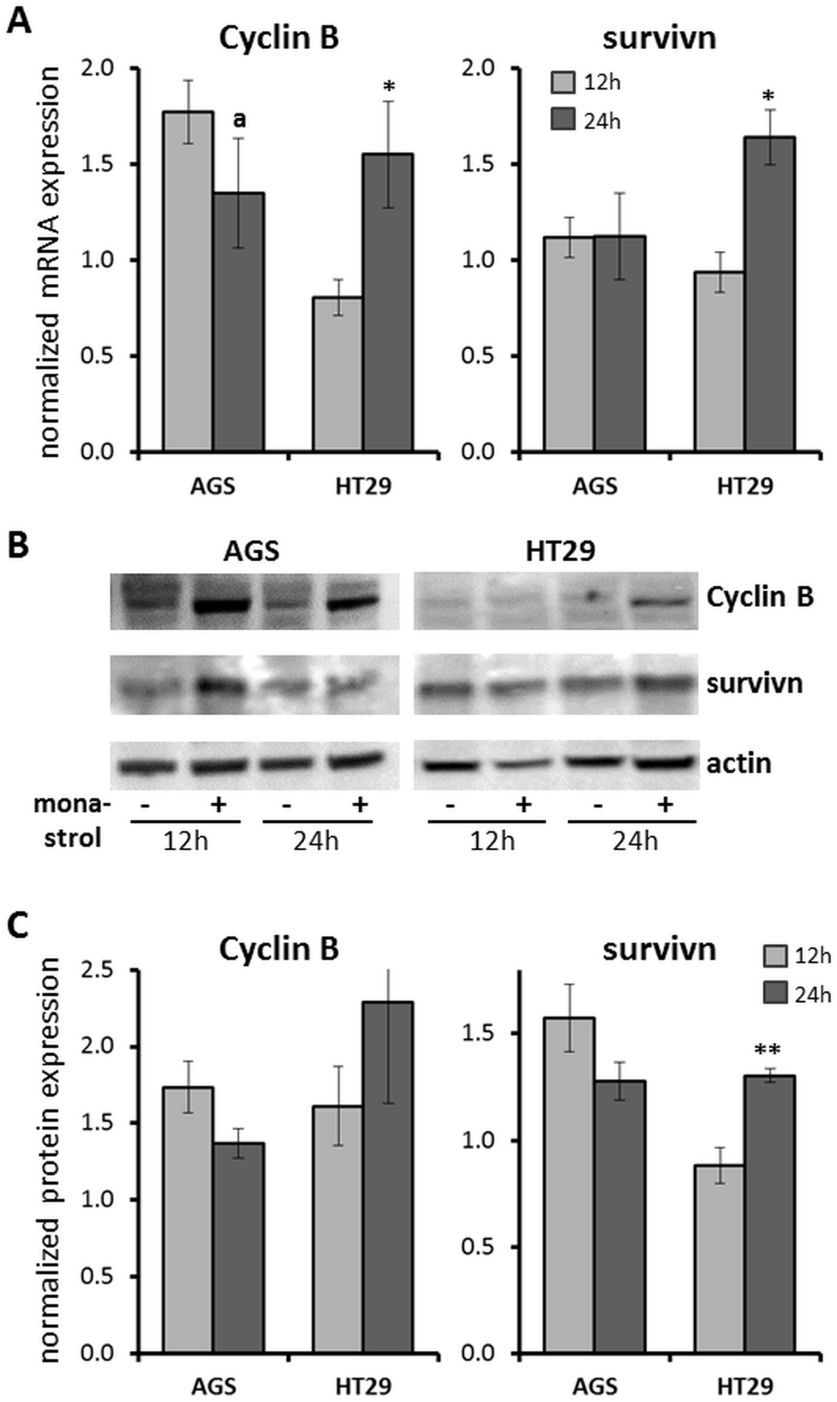
Expression levels of the mitotic cyclin B and survivin proteins. HT29 and AGS cells were treated with monastrol for 12h and 24h and processed for mRNA analysis by RT PCR (A) and protein level analysis by WB (B and C). 150μM and 100μM monastrol were used for HT29 and AGS cells, respectively. In A and C, columns and bars represent average and SEM values for 3–4 independent experiments. In each experiment, levels of cyclin B (left) and survivin (right), indicated at the top, in the presence of monastrol were normalized to the control values obtained with DMSO only. *P<0.05; **P<0.01; a—0.05<P<0.1 compared to 12h of monastrol treatment. (B) Representative WB analysis of cyclin B and survivin protein expression in AGS (left) and HT29 (right) cells treated with monastrol for 12h and 24h, indicated on the bottom. Equal protein loading was follow by the expression of actin (lower panels). (+) Monastrol; (-) DMSO.

Down-regulation of the anti-apoptotic protein survivin was previously shown to increase mitotic slippage in the presence of spindle damage [52]. We thus hypothesized that during exposure to monastrol, the expression of survivin will differ between monastrol-sensitive and -resistant cells. To test this hypothesis, we compared mRNA and protein levels of survivin in monastrol-resistant HT29 cells and monastrol-sensitive AGS cells (Fig 3). To allow measurement of changes in protein level before apoptosis [30], we treated cells with monastrol for shorter times, 12h and 24h. To assess the mitotic status of the examined cells, we followed mRNA (Fig 3A) and protein (Fig 3B and 3C) levels of the mitotic protein cyclin B. We found that following 24h treatment with monastrol HT29 cells exhibit increased levels of cyclin B compared to 12h of treatment, indicating that they are arrested in mitosis (Fig 3). On the other hand, in AGS cells, cyclin B levels were already increased at 12h of treatment with monastrol and cyclin B levels exhibited a tendency to decline at 24h, 0.05<P<0.1 (Fig 3). This suggests that treatment with monastrol for 24h already causes partial mitotic slippage in AGS cells while the HT29 cells maintain mitotic arrest. Examination of survivin mRNA (Fig 3A) and protein (Fig 3B and 3C) levels revealed that in HT29 cells’ levels of survivin increased at 24h treatment with monastrol, suggesting that survivin is involved in maintaining the mitotic arrest. On the other hand, in the AGS cells mRNA levels of survivin remained unchanged at 24h compared to 12h treatment with monastrol. Moreover, in the AGS cells, average protein levels of survivin were lower at 24h compared to 12h of monastrol treatment, although this difference did not reach statistical significance (Fig 3B and 3C, right panel). These results suggest that elevated levels of survivin in a particular cell-line are correlated with the resistance of this cell-line to monastrol treatment.

**Fig 4:**
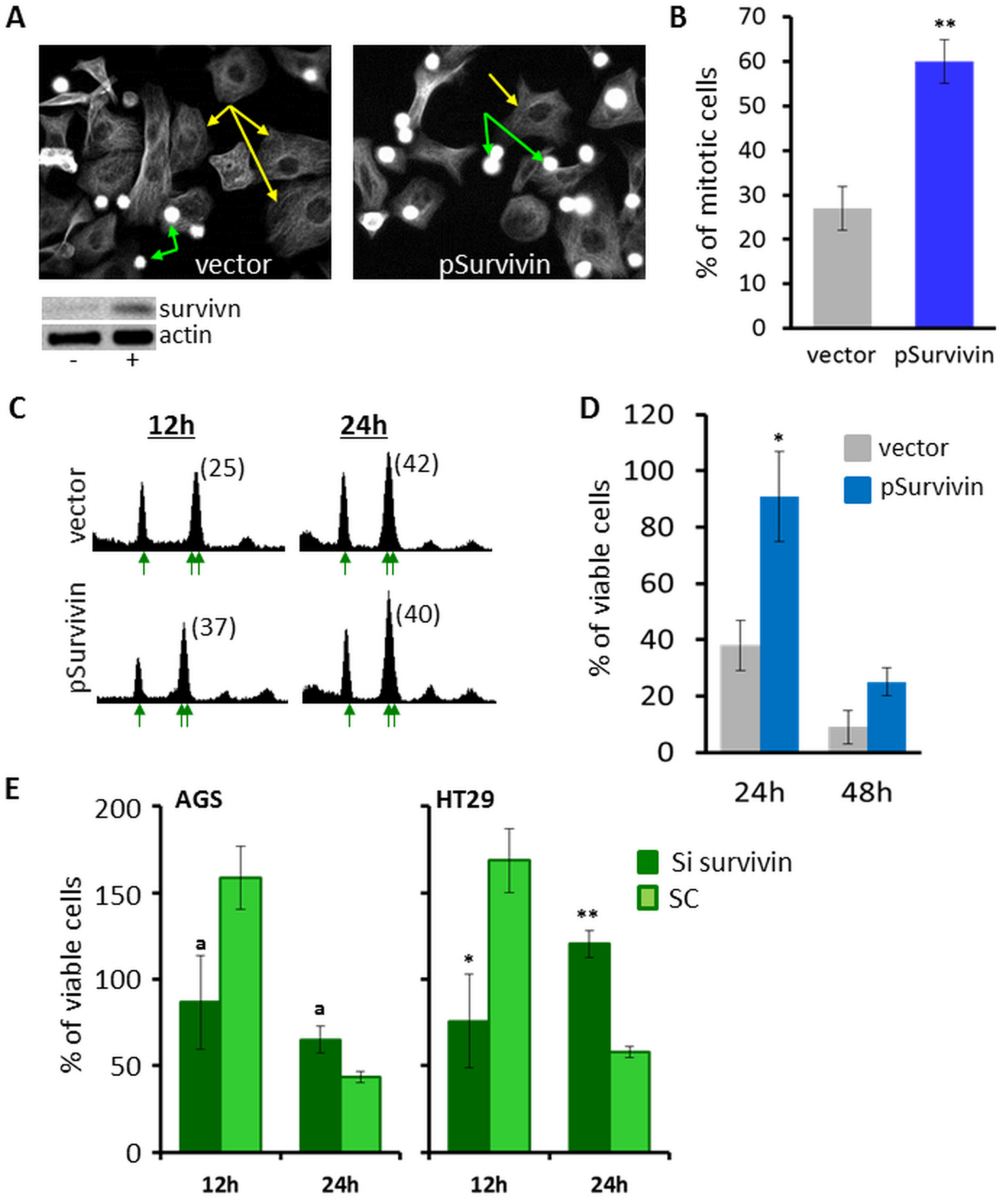
Elevated expression of survivin induces mitotic arrest and increases cell viability in the presence of monastrol. (A–D) AGS cells were stably transfected with a plasmid expressing GFP (vector) or a plasmid that encodes for overexpression of survivin-GFP (pSurvivin). Cells were treated with 100μM monastrol and processed for microtubule immunostaining (A and B), cell-cycle distribution analysis (C), and viability test (D). (A) Representative images of microtubule cytoskeleton in AGS cells carrying an empty vector or pSurvivin, indicated at the bottom. Cells were treated with 100μM monastrol for 24h. G1-like cells (yellow arrows) and mitotic cells (green arrow) are observed. Since the mitotic cells are round, their focal plane is different from that of the flat G1-like cells. Images in (A) are focused on the G1 cells and therefore the monoasters in mitosis are not seen in these images. Consequently, mitotic cells appear as bright spheres (green arrows). Bottom: survivin WB analysis of proliferating AGS cells in the presence (+) or absence (-) of the pSurvivin overexpression plasmid. (B) Quantification of % of mitotic cells based on images such as shown in (A). Gray—vector only; blue—pSurvivin. Columns and bars represent averages and SEM of three independent experiments in which a total of 200–300 cells were counted. **P<0.01, compared to vector only. (C) Stably-transfected AGS cells were treated with monastrol for 12h and 24h (indicated on top) and processed for cell-cycle phase distribution analysis by flow cytometry. Single and double arrows represent peaks with 2n and 4n DNA content, respectively. Numbers in parentheses represent the percentage of cells with double (4n) DNA content. (D) Viability of stably transfected cells in the presence of 100μM monastrol for 24h and 48h. Gray—vector only; Blue—pSurvivin. The number of viable cells was determined by XTT assay for mitochondrial activity. Percent of viable cells was calculated relative to control experiments with DMSO only. Columns and bars represent average and SEM of 3 independent experiments. *P<0.05, compared to vector only. (E) AGS and HT29 cells were transiently transfected with survivin (dark green) or scrambled (SC, light green) Si RNA sequence. 12h following transfection AGS and HT29 cells were treated with 100 and 150μM monastrol, respectively, for 12h and 24h (indicated on the bottom). Viability of cells was determined by Trypan blue. Percent of viable cells was calculated relative to control experiments with DMSO only. Columns and bars represent average and SEM of 2–4 independent experiments preformed in triplicates. *P<0.05; **P<0.01; a—0.05<P<0.1, compared to scrambled Si RNA sequence.

To examine whether elevated expression levels of survivin directly maintain sustained mitotic arrest, we transfected the monastrol-sensitive AGS cells with a plasmid encoding for wild-type survivin protein fused to GFP (pSurvivin, [45]), and created a poly-clonal stably transfected AGS cell-line (see Materials and Methods). GFP-expressing vector was used as a control. AGS cells expressing the pSurvivin plasmid exhibited 5.2±0.9 (SEM, n = 4) fold increase in survivin expression (Fig 4A, bottom). Following the generation of stably-transfected cell-lines, cells were treated with 100μM monastrol and processed for tubulin immunostaining, viability, and cell-cycle distribution analysis (Fig 4A–4D). We found that following 24h treatment with monastrol, the percentage of mitotic cells arrested with monoasters significantly increased in survivin-expressing cells compared to AGS cells expressing vector only (Fig 4A and 4B). These data indicate that overexpression of survivin increases the ability of the AGS cells to maintain mitotic arrest in the presence of spindle damage induced by monastrol. Analysis of the cell-cycle phase distribution indicates that following 12h treatment with monastrol, the percentage of survivin-expressing AGS cells with double (4n) DNA content is significantly increased compared to cells expressing vector only (Fig 4C, 12h), indicating that survivin induces sustained mitotic arrest. Importantly, following 24h treatment with monastrol we found no difference in percentage of cell population with 4n DNA content between the cells that express survivin and those that express vector only (Fig 4C, 24h). Yet, following 24h treatment with monastrol, there was a significant increase in the number of cells with monoasters in survivin-expressing cells, indicating the cells are in mitotic arrest, compared to cells expressing vector only, which underwent mitotic slippage and were destined to cell death (Fig 4A and 4B). Thus, our data indicate that in the absence of survivin overexpression, AGS cells undergo mitotic slippage and cell death following treatment with monastrol, while overexpression of survivin induces mitotic arrest, likely rescuing the cells. In addition, consistent with the suggested anti-apoptotic function of survivin [45, 53], we found that overexpression of survivin in AGS cells increased the viability of these cells following 24h and 48h treatment with monastrol (Fig 4C).

To further examine the linkage between cell viability under monastrol treatment and expression of survivin, we partially silenced survivin expression by Si RNA (Fig 4E). Scrambled RNA sequence served as a control. Examination of mRNA levels of survivin by RT PCR confirmed that while transient transfection of scrambled RNA sequence caused no change in the mRNA levels of survivin, transfection with survivin Si RNA caused ~ 70% and 50–60% decrease in survivin mRNA levels in AGS and HT29 cells, respectively (not shown). We found that following 12h of exposure to monastrol, partial silencing of survivin expression caused a decrease in viability of AGS and HT29 cells by 50–60%, compared to cells transfected with the scrambled RNA sequence (Fig 4E). While the monastrol-sensitive AGS cells showed only a tendency (0.05<P<0.1), the monastrol-resistant HT29 cells showed a clearly significant decrease in viability following partial surviving silencing (P<0.05), indicating that survivin is required for viability and resistance to monastrol. Following 24h of treatment with monastrol, the viability of the monastrol-sensitive AGS cells was further reduced in cells transfected with survivin Si or scrambled RNA (Fig 4E). In the monastrol-resistant HT29 cells, partial silencing of survivin expression induced an increased viability compared to the scrambled RNA sequence, indicating that there are adaptation mechanisms in these cells to partially reduced levels of survivin. Taken together, our data indicate that increasing levels of survivin during mitotic arrest are linked to cell viability, which is at least partially related to its ability to induce prolonged mitotic arrest under treatment with monastrol.

## Discussion

Studies over the last decade have indicated that when cells are treated with anti-mitotic drugs such as kinesin-5 specific inhibitors, two principal scenarios can occur: cells can either maintain mitotic arrest and die by mitotic apoptosis [30, 31], or proceed to the next G1 stage by mitotic slippage and undergo apoptosis [33, 34]. In the latter case, cells rapidly die of apoptosis caused by the "tetraploidy checkpoint" [33, 35]. By this scenario, cells that undergo mitotic slippage more readily in the presence of monastrol will be subjected to apoptosis induced by the tetraploidy checkpoint and thus be more sensitive to monastrol. However, the tetraploidy checkpoint was found to be dependent on the tumor suppressor protein p53 [51, 54]. Thus, cells are expected to be more sensitive to monastrol if they undergo mitotic slippage and express a wild-type p53. Indeed the cell-lines examined in this study express both versions of the p53 protein: p53 is wild-type in AGS [55], HepG2 [56], and LoVo [57] cell lines, while it is mutated in HT29 [58] and Du145 [49] cells. Our finding that the rank of sensitivity to monastrol is AGS>HepG2>Lovo>Du145≥HT29 supports the idea that cells will be more sensitive to the kinesin-5 inhibitors if they tend to undergo mitotic slippage and carry wild-type p53 allele. The tendency to undergo mitotic slippage per se is not directly dependent on the p53 status since the LoVo cells that carry WT p53 [57] undergo mitotic slippage to the same degree as the HT29 and Du125 cells, which carry a mutated p53 [49, 58] (Fig 2B and 2C). Nevertheless, increased cell death occurs in the LoVo cells when WT p53 allows activation of the "tetraploidy checkpoint".

When mitotic slippage occurs in p53 mutated cells, cells proceed with proliferation without properly dividing DNA. *In vivo*, this may result in accumulation of mutations that reduce cytotoxic/anti-cancer activity of kinesin-5 inhibitors [22, 27, 39]. Thus, one of the strategies to increase the efficiency of anti-mitotic anti-cancer drugs may be to induce prolonged mitotic arrest in cancers expressing mutated versions of p53. Previous studies have indicated that when survivin is down regulated, cells undergo mitotic slippage in the presence of spindle damage [52]. Here we show that overexpression of wild-type survivin induces a prolonged mitotic arrest when spindle damage is inflicted by a kinesin-5 inhibitor, monastrol (Fig 4A, 4B and 4C). Prolonged mitotic arrest induced by survivin can increase the “window of opportunity” for mitosis-dependent apoptosis to occur when cells are treated with kinesin-5 inhibitors. This is likely to be applicable in preventing mitotic slippage in p53 mutated cancers. In cancers expressing WT p53 treated by kinesin-5 inhibitors, down-regulation of survivin is likely to be a preferred strategy for treatment, inducing mitotic slippage and apoptotic death by the tetraploidy checkpoint.

Survivin is a multi-functional protein that was shown to have an anti-apoptotic role as well as multiple mitotic functions [42, 43]. As such, it was examined as a possible target for anticancer treatment [40, 41]. Based on our results, we suggest that analysis of the p53 status of a particular tumor and combined treatment with kinesin-5 inhibitor and modulation of survivin expression, either up or down, may improve the efficacy of the anti-cancer treatment.
